# Eco-Friendly Materials Obtained by Fly Ash Sulphuric Activation for Cadmium Ions Removal

**DOI:** 10.3390/ma13163584

**Published:** 2020-08-13

**Authors:** Gabriela Buema, Nicoleta Lupu, Horia Chiriac, Tiberiu Roman, Marieta Porcescu, Gabriela Ciobanu, Daniela Vasilica Burghila, Maria Harja

**Affiliations:** 1 National Institute of Research and Development for Technical Physics, 47 Mangeron Boulevard, 700050 Iasi, Romania; gbuema@phys-iasi.ro (G.B.); nicole@phys-iasi.ro (N.L.); hchiriac@phys-iasi.ro (H.C.); troman@phys-iasi.ro (T.R.); porcescu@phys-tuiasi.ro (M.P.); 2 Integrated Center of Environmental Science Studies in the North Eastern Region—CERNESIM, “Alexandru Ioan Cuza” University of Iasi, Carol I nr. 11 Blvd., 700506 Iasi, Romania; 3 Faculty of Chemical Engineering and Environmental Protection, “Gheorghe Asachi” Technical University of Iasi, 73 Prof.dr.doc. Dimitrie Mangeron Street, 700050 Iasi, Romania; gciobanu@tuiasi.ro; 4 Faculty of Land Reclamation and Environmental Engineering, University of Agronomical Sciences and Veterinary Medicine of Bucharest, 59 Mărăști Blvd., 011464 Bucharest, Romania; dburghila@gmail.com

**Keywords:** acidic fly ash modification, adsorption, cadmium ions, isotherms, kinetic models

## Abstract

Wastes are the sustainable sources of raw materials for the synthesis of new adsorbent materials. This study has as objectives the advanced capitalization of fly ash, by sulphuric acid activation methods, and testing of synthesized materials for heavy metals removal. Based on the previous studies, the synthesis parameters were 1/3 s/L ratio, 80 °C temperature and 10% diluted sulphuric acid, which permitted the synthesis of an eco-friendly adsorbent. The prepared adsorbent was characterized through SEM, EDX, FTIR, XRD and BET methods. Adsorption studies were carried out for the removal of Cd^2+^ ions, recognized as ions dangerous for the environment. The effects of adsorbent dose, contact time and metal ion concentrations were studied. The data were tested in terms of Langmuir and Freundlich isotherm and it was found that the Langmuir isotherm fitted the adsorption with a maximum adsorption capacity of 28.09 mg/g. Kinetic data were evaluated with the pseudo-first-order model, the pseudo-second-order model and the intraparticle diffusion model. The kinetics of cadmium adsorption into eco-friendly material was described with the pseudo-second-order model, which indicated the chemisorption mechanism.

## 1. Introduction

The presence of different pollutants in water that are characterized by their non-biodegradable nature can affect the environment and humans’ health [1,2,3]. Due to pollutants, heavy metals occur in a significant number of places; this type of pollutant represents one of the biggest environmental problems [4,5]. Consequently, a sustainable solution must be found in order to solve this problem. Out of all possible removal methods, adsorption is one of the most applied techniques for treating contaminated waters due to some advantageous criteria, such as high performance, low cost, easy operation, wide pH range, etc. [6,7]. Out of all the available adsorbents, activated carbon and zeolites can be used; for low cost adsorbents, however, fly ash, modified ash, agricultural wastes, etc., are considered [7,8,9].

As a result of coal combustion, fly ash is found in large quantities and it has been reported that this fact causes serious environmental problems [10,11]; therefore, it must be addressed [12,13]. Fly ash is rich in aluminosilicate and includes polymeric minerals and inorganic oxide material [14,15]. The use of ash as an adsorbent for the retention of heavy metals is a widely studied field [16,17,18,19]. To increase the adsorption capacity, activation of the material surface by using acid or base chemical treatment is recommended. Studies have shown that the modification of ash can be done in different ways [16,20,21,22,23]. By applying one of these methods, various zeolitic structures are obtained: Analcime (A), Chabazite (Cha), Cancrinite (Can), NaP1, Na-Y, Sodalite (S), Fau (Faujasite) [11,24].

The new materials obtained by modification have been continuously investigated in recent decades regarding the treatment of wastewater [8,17,20,25,26,27,28]. The literature presents different adsorbents based on modified fly ash for cadmium adsorption [25]. Unfortunately, there is no data available regarding the adsorption of Cd^2+^ ions into acid activated fly ash. Therefore, a detailed study regarding the utilization of this material by evaluating the adsorption process is necessary.

The research was performed in May–June 2020 at The National Institute of Research and Development for Technical Physics, Romania. It is the continuation of the experiments where the adsorption capacity of modified fly ash with sodium hydroxide for the removal of Cd^2+^ ions was tested [25]. In this study, for the first time, fly ash treated with H_2_SO_4_ was used for adsorption of Cd^2+^ ions from aqueous solutions. The equilibrium data were modeled using Langmuir and Freundlich isotherm models; the kinetics data were designed based on the pseudo-first order, pseudo-second order and intraparticle diffusion models. A characterization of synthesized adsorbent is also included in this paper.

## 2. Materials and Methods

### 2.1. Materials

The fly ash was supplied by CET II Holboca, a thermo-electric power plant from Iasi, North Romania. The fly ash was a priori characterized and the properties have been published in previous papers [29]. Of the properties studied in this research, the important ones are: Particle size 0.01–100 μm; chemical composition: SiO_2_—58.62%; Al_2_O_3_—16.98%; Fe_2_O_3_—8.78%; CaO—8.41%; MgO—1.29%; and other components also up to 0.5% [29].

The chemical reagents were analytical grade and were used as received from Sigma Aldrich.

Cd^2+^ was the heavy metal studied in this research. It is recognized as one of the most toxic heavy metals that enter into water due to various activities, so its removal is required [25].

An aqueous solution of cadmium nitrate was prepared by dissolving a quantity of Cd(NO_3_)_2_ 4H_2_O into 1 L of water in order to obtain an initial solution of 1000 mg/L. The working solutions of 50–320 mg/L were prepared by diluting the stock solution of Cd(NO_3_)_2_ 4H_2_O (1000 mg/L).

For synthesized material, SEM and EDAX analyses were carried out using Vega Tescan LMH II (Brno–Kohoutovice, Czech Republic) and Bruker EDAX with XFlash detector (Brno, Czech Republic), FTIR analysis was realized with the Bruker Vertex 60 device (Ettlingen, Germany), specific surface area was calculated with the BET method using low-temperature nitrogen adsorption/desorption data obtained via Quantachrome instruments, the Nova 2200e model (Graz, Austria), and X-ray diffraction (XRD) patterns were recorded using an Advance D8-Bruker X-ray diffractometer with Cu-Kα radiation (Malvern, United Kingdom).

### 2.2. Adsorbent Synthesis

The fly ash, without any processing, was mixed with the sulfuric acid solution. The acid quantity was determined by the stoichiometry of reactions between the iron, aluminum and calcium oxides, assuring a 5–10% excess. A quantity of 200 g of fly ash and 500 mL of 10% sulfuric acid solution was added to the reactor [30], the mixture was stirred mechanically at 200 rot/min, 80 °C, and the curing time was 2 h. After that time, the FA/H_2_SO_4_ was cooled at ambient temperature for 18 h to ensure the crystallization of the new products and was filtered and dried at 70 °C until the weight was constant. The prepared solid was stored in a drying vessel and used as FA/H_2_SO_4_.

### 2.3. Experimental Procedure

The adsorption experiments for the evaluation of the material adsorption capacities, including the study of the influence of adsorbent dose, initial concentration, and contact time, were performed as follows:

FA/H_2_SO_4_ dose influence: Different quantities of adsorbent (8–20 g/L) were added to 25 mL of Cd^2+^ concentration of 70 mg/L at pH 5.0 ± 0.1, room temperature and contact time 24 h (for reaching equilibrium), with intermittent stirring;

Initial concentration influence: A dose of 8 g/L of adsorbent was added to 25 mL of Cd^2+^ concentration (50–320 mg/L), pH 5.0 ± 0.1, room temperature, and contact time 24 h, with intermittent stirring;

Contact time influence: A dose of 8 g/L of adsorbent was added to 25 mL of Cd^2+^ concentration of 70 mg/L, pH 5.0 ± 0.1, temperature 24.6 °C, contact time 5–120 min, with intermittent stirring.

The Cd^2+^ concentration in the supernatant solution was measured using the UV-visible spectrophotometry method at 576 nm (Perkin Elmer Lambda 35 UV/VIS spectrophotometer–Llantrisant, United Kingdom). All the results were carried out in triplicate.

The amount of equilibrium adsorption capacity was evaluated according to the equation [31]:

(1) $$q=\frac{\left(C_{0}-C_{e}\right)V}{m}$$

where $C_{0}$ and $C_{e}$ are the initial and equilibrium Cd^2+^ concentrations (mg/L), q is the amount of Cd^2+^ adsorbed onto FA/H_2_SO_4_ (mg/g), *V* is the volume of solution (*L)* and *m* is the quantity of FA/H_2_SO_4_ (g).

The adsorption capacity at different time intervals (5–120 min) was calculated with Equation (2):

(2) $$q_{t}=\frac{\left(C_{0}-C_{t}\right)V}{m}$$

where $C_{t}$ is Cd^2+^ concentrations at different time intervals (mg/L), $q_{t}$ is the amount of Cd^2+^ adsorbed onto FA/H_2_SO_4_ at time intervals = 5–120 min, *V* is the volume of solution (L), and *m* is the quantity of FA/H_2_SO_4_ (g).

## 3. Results

### 3.1. Characterization of Adsorbent

The characterization of the FA/H_2_SO_4_ adsorbent was performed by SEM, EDX, FTIR, XRD and BET points of view. Moreover, SEM, EDX, FTIR and XRD analyses for FA/H_2_SO_4_ after Cd^2+^ adsorption, noted as FA/H_2_SO_4_+Cd^2+^, were included.

#### 3.1.1. SEM Analysis

**Figure 1:**
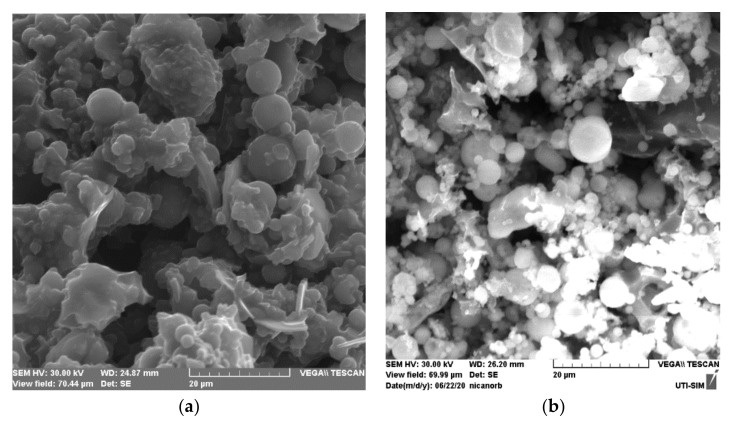
SEM images for FA/H_2_SO_4_ (**a**) and FA/H_2_SO_4_+Cd^2+^ (**b**), at a resolution of 20 µm.

Figure 1 shows SEM analyses for FA/H_2_SO_4_ and for FA/H_2_SO_4_+Cd^2+^. SEM analysis (Figure 1a) indicates that the surface of FA/H_2_SO_4_ material contains spherical shapes with different sizes and smooth surfaces, similar to unmodified fly ash [4,24,32].

The white patches that can be seen in Figure 1b can be explained by the presence of Cd^2+^ on the surface of FA/H_2_SO_4_ material. Moreover, this fact could be observed in the case of U(VI) uptake onto the adsorbent based on ash [16].

It can be observed that the widths of FA were decomposed into small particles. This is a result of sulphuric acid activation, which can destroy the whole structure of FA by the reaction of acid with the alkali and oxide compounds (CaO, MgO, Al_2_O_3_, Fe_2_O_3_, etc.). In light of Figure 1b, it can be observed that FA/H_2_SO_4_ has a relative quantity of the smaller particle sizes.

#### 3.1.2. EDX Analysis

**Figure 2:**
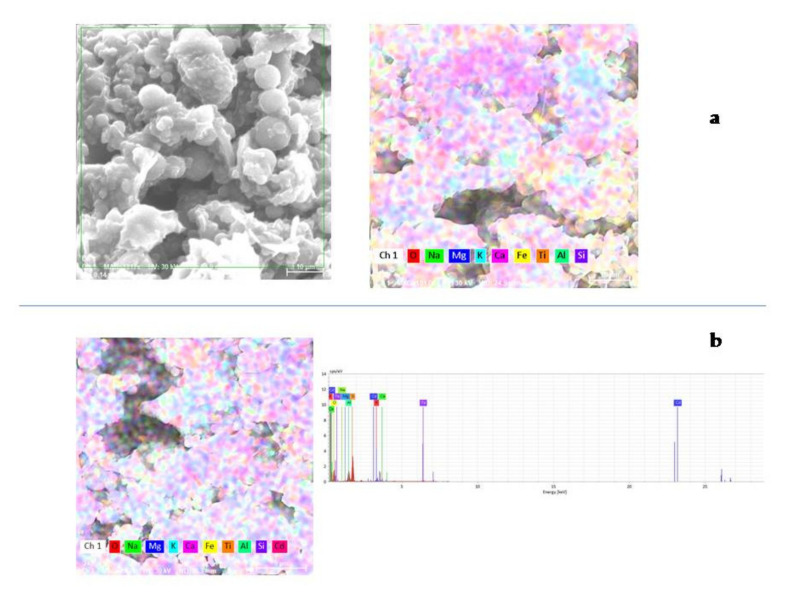
Elemental analysis by EDX for FA/H_2_SO_4_ (**a**); FA/H_2_SO_4_+Cd^2+^ (**b**).

Furthermore, EDX analysis for FA/H_2_SO_4_ was recorded in order to establish the elemental composition. The results are presented in Figure 2a and Table 1.

EDX analysis of FA/H_2_SO_4_ reveals the peaks for O, Si and Al elements and smaller peaks for elements such as Ca, Fe, K, Mg, Na and Ti.

Moreover, the EDX spectrum of the FA/H2SO4+Cd^2+^ sample is presented in Figure 2b and Table 2. The adsorption of Cd^2+^ was confirmed by the peak of Cd in the EDX spectrum.

#### 3.1.3. FTIR Analysis

**Figure 3:**
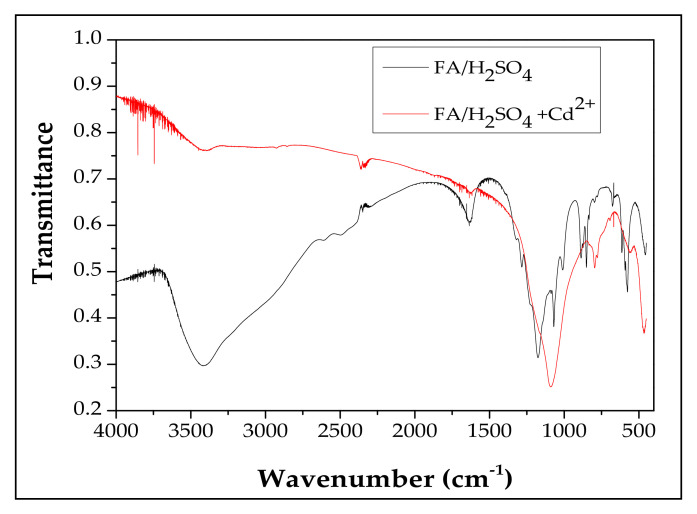
FTIR spectra of FA/H_2_SO_4_ and FA/H_2_SO_4_ + Cd^2+^.

FTIR analysis was employed to establish the functional groups that could provide information concerning the structure of the adsorbent. In Figure 3 the FTIR spectra for FA/H_2_SO_4_ and FA/H_2_SO_4_+Cd^2+^ can be seen.

Regarding the FA/H_2_SO_4_ sample, the new bands found at 1629 cm^−1^, 888 cm^−1^ and 848 cm^−1^ in comparison with unmodified fly ash [29] can be explained by the presence of S-O on the unmodified fly ash surface. Based on the results, it can be concluded that the synthesis was successfully done.

On the other hand, after Cd^2+^ adsorption there was a shift in the bands between 1700 cm^−1^ and 400 cm^−1^, which proves the adsorption process of Cd^2+^ on the FA/H_2_SO_4_ sample [33,34].

#### 3.1.4. XRD Analysis

**Figure 4:**
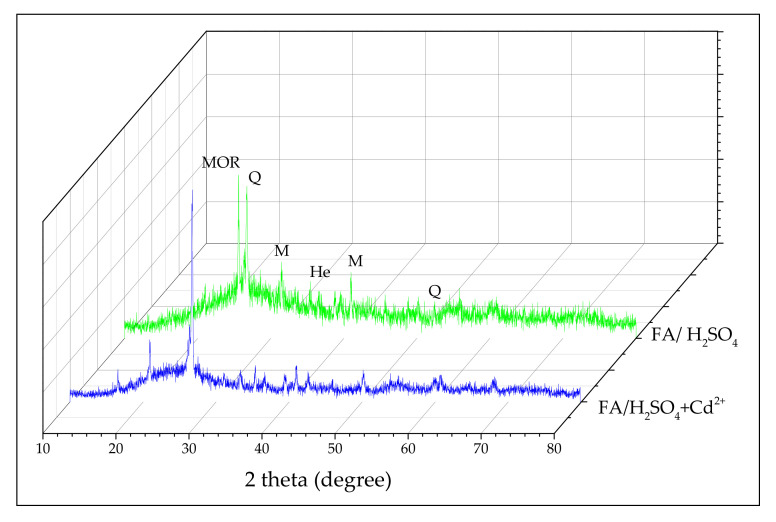
X-ray diffraction patterns of FA/H_2_SO_4_ and FA/H_2_SO_4_ +Cd^2+^.

Figure 4 represents the comparison of XRD patterns between FA/H_2_SO_4_ and FA/H_2_SO_4_+Cd^2+^.

One significant observation is that the results of XRD analysis revealed that quartz (Q) presents the highest intensity—mullite (M) and hematite (He); the mainly crystalline phases of the unmodified fly ash [26] can also be identified in FA/H_2_SO_4_. Additionally, FA/H_2_SO_4_ shows a prominent peak at 2 theta = 25.63° that can be attributed to modernite (MOR)[Na_8_(H_2_O)_24_] [Si_40_Al_8_O_96_] [35,36]. In the case of FA/H_2_SO_4_+Cd^2+^ the peaks can be observed in minor intensity. On the other hand X-ray diffraction patterns indicate that a large part of the amorphous silica is present in the tested samples.

#### 3.1.5. BET Analysis

**Figure 5:**
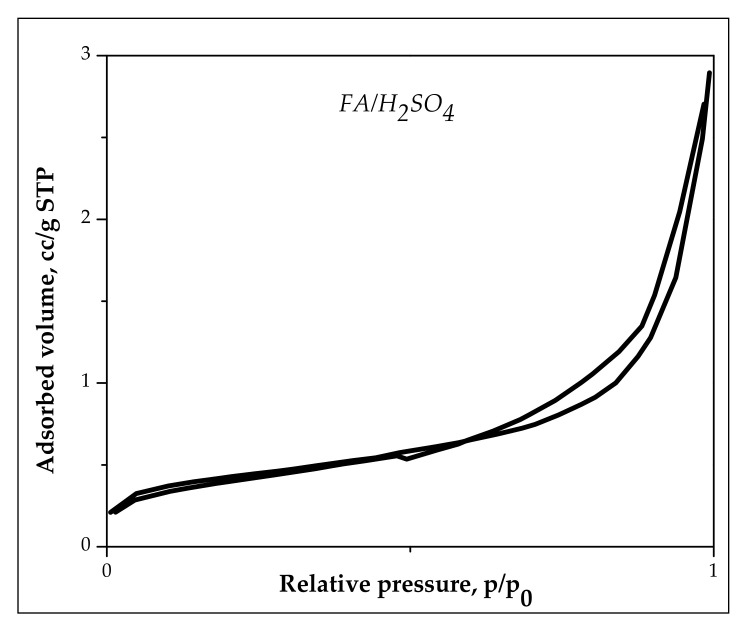
Nitrogen adsorption isotherm at 77 K on FA/H_2_SO_4_.

The N_2_ adsorption–desorption isotherm is shown in Figure 5.

The BET results shows that the specific surface area of FA/H_2_SO_4_ is 1.53 times higher compared with FA [8]; the specific area for FA/H_2_SO_4_ is 10.71 m^2^/g. In addition, the modification of chemical components (alkali substance, metallic oxides) by acid activation increases the pore volume and decreases the average pore diameter. The total pore volume was 0.068 cm^3^/g for FA/H_2_SO_4_, a comparative increase compared to 0.042 cm^3^/g for raw fly ash. The diameters of the pores were smaller than 18.7 nm.

All physical and chemical changes of FA are the essential features of the sulphuric acid-based activation of FA/H_2_SO_4_. To determine whether a material can be proposed as an adsorbent for the retention of heavy metals and also for establishing the optimal adsorption experimental conditions, at least two parameters should be evaluated.

### 3.2. Effect of FA/H_2_SO_4_ Dose in Cd^2+^ Adsorption

Studies have proved that the recommended pH for the removal of Cd^2+^ must be below 6.5, because at a higher pH value the precipitation of Cd(OH)_2_ in solution can occur [25,37].

**Figure 6:**
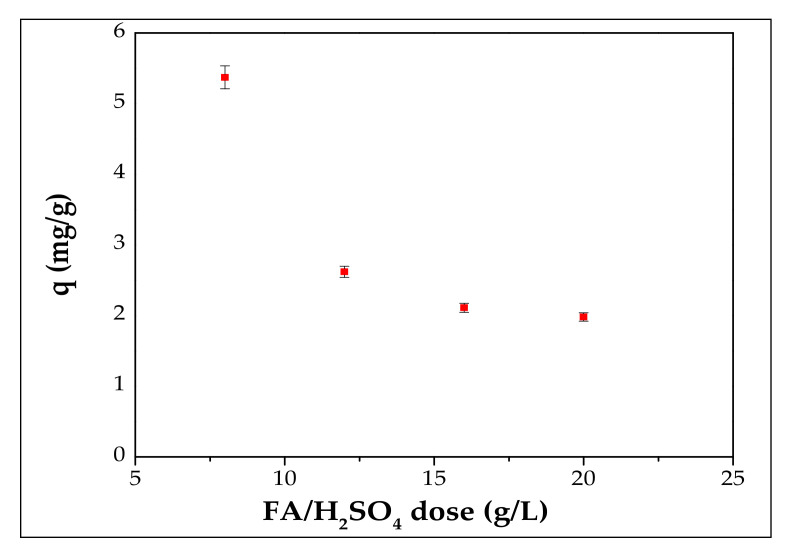
Effect of FA/H_2_SO_4_ dose in Cd^2+^ adsorption.

Adsorbent dose should be a parameter studied in the adsorption technique of different pollutants from wastewaters since it has an important role in terms of process economy [38,39]. Determining the optimal dose of the adsorbent was carried out by increasing the FA/H_2_SO_4_ dose from 0.2 g/25 mL to 0.5 g/25 mL at an initial concentration of 70 mg/L. The effect of the adsorbent dose through adsorption capacity is presented in Figure 6.

Furthermore, the effect of adsorbent dose on the adsorption capacity indicated that, as the adsorbent dose increases from 8 to 20 g/L, the adsorption capacity decreases from 5.37 mg/g to 1.98 mg/g. It was found that these results are consistent with the published literature [34]. The explanation could be that at higher adsorbent dose, aggregation of particles takes place. Therefore, for all further experiments adsorbent dose was fixed at 8 g/L solution.

### 3.3. Effect of Initial Concentration and Adsorption Isotherm

**Figure 7:**
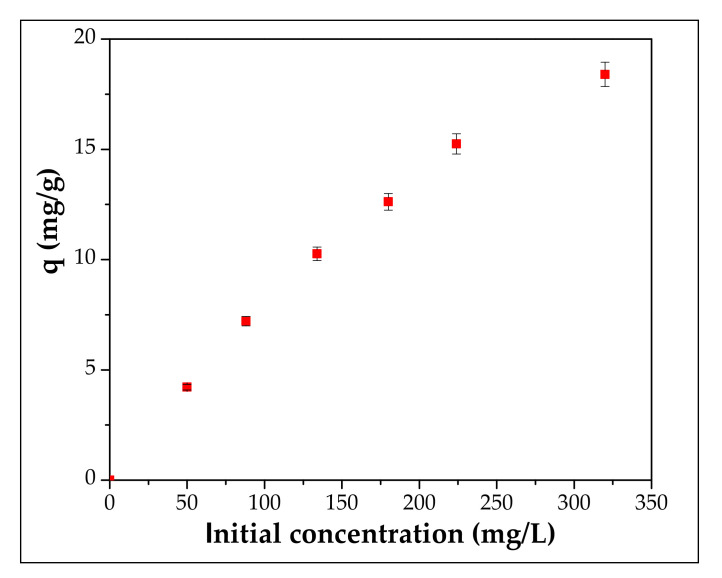
Effect of initial concentration on the adsorption of Cd^2+^ onto adsorbent.

For the optimization of this parameter, different concentrations of Cd^2+^ (50–320 mg/L) were applied and the results are presented in Figure 7.

It is obvious that the initial concentration has an impact on the adsorption capacity; thus, at low concentrations small adsorption capacity values are obtained, and as the concentration increases, the adsorption capacity also increases. For example, at an initial Cd^2+^ concentration of 50 mg/L the adsorption capacity is equal with 4.22 mg/g, while at 320 mg/L initial Cd^2+^ concentration, the adsorption capacity increased by approximately 78%. Experimental results at Cd^2+^ concentration range of 50–320 mg/L were explained based on Langmuir and Freundlich adsorption isotherm model points of view and the corresponding parameters were obtained accordingly.

**Figure 8:**
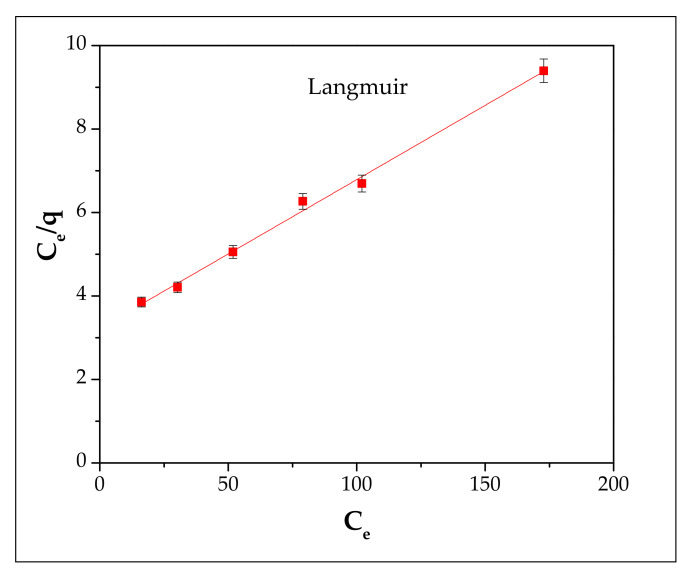
Langmuir isotherm plot of Cd^2+^ adsorption on FA/H_2_SO_4_.

**Figure 9:**
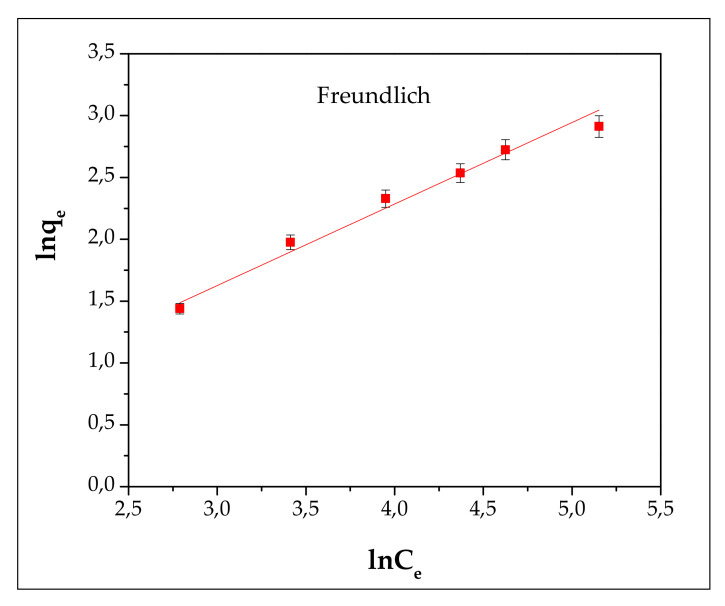
Freundlich isotherm plot of Cd^2+^ adsorption on FA/H_2_SO_4_.

A plot of C_e_/q vs. C_e_ (Langmuir) and lnq_e_ vs. lnC_e_ (Freundlich) enables the determining of the coefficients (Figure 8 and Figure 9 and Table 3).

According to the R^2^ value of the Langmuir (0.9956) and Freundlich (0.9806) isotherms, it can be highlighted that the Langmuir model is suitable for describing the adsorption process of Cd^2+^ onto FA/H_2_SO_4_.

### 3.4. Effect of Contact Time and Kinetic Models

It is very common in the literature that the adsorption takes place in two stages: (a) An initial stage that includes the fast adsorption in the first minutes from the beginning of the adsorption process and (b) a final stage that include the slow adsorption.

**Figure 10:**
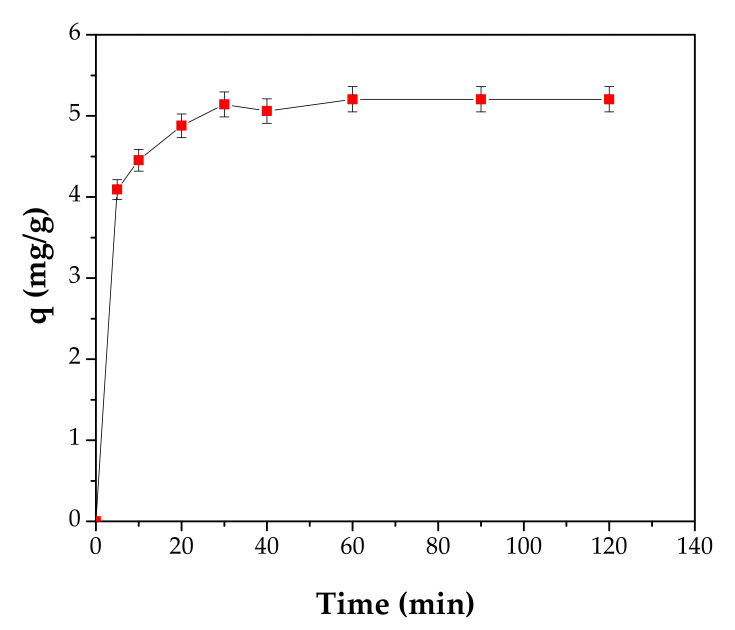
Effect of contact time on Cd^2+^ adsorption, C_0_ 70 mg/L, pH 5 and 8 g/L adsorbent dose.

The adsorption capacity was investigated as a function of contact time at 70 mg/L initial Cd^2+^ concentration, pH 5.0 and an adsorbent dose of 8 g/L. The kinetic of adsorption of Cd^2+^ was carried out by withdrawing and analyzing the samples until the adsorption capacities became closer. The results are presented in Figure 10.

From Figure 10 it can be noted that the adsorption capacity increased quickly from 4.09 to 5.2 mg/g as the contact time increased from 5 to 60 min. By increasing the contact time to 120 min, a plateau is obtained. This fact could be explained as follows: The increase of the contact time determines the filling of the pores and thus the rate becomes slower.

It might be concluded that a contact time of 60 min could be considered sufficient for 60% adsorption under the experimental conditions.

**Figure 11:**
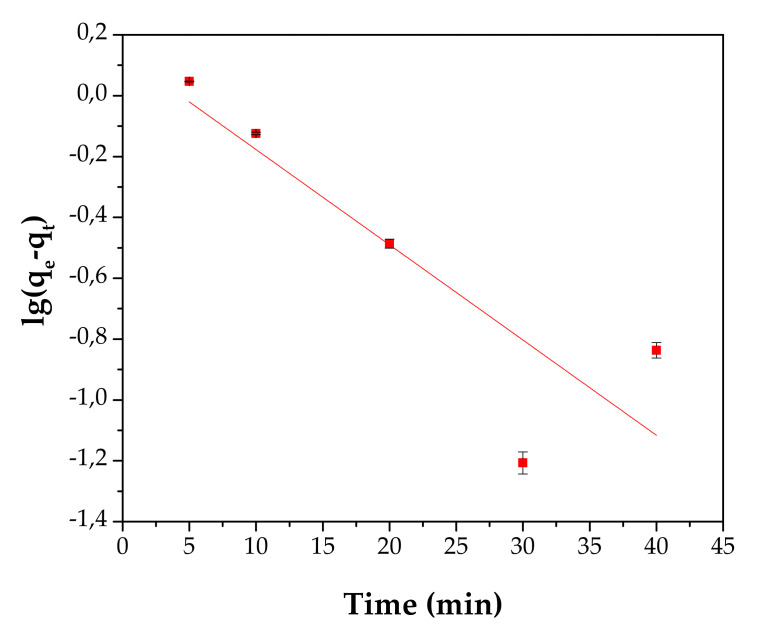
Pseudo-first-order kinetics.

**Figure 12:**
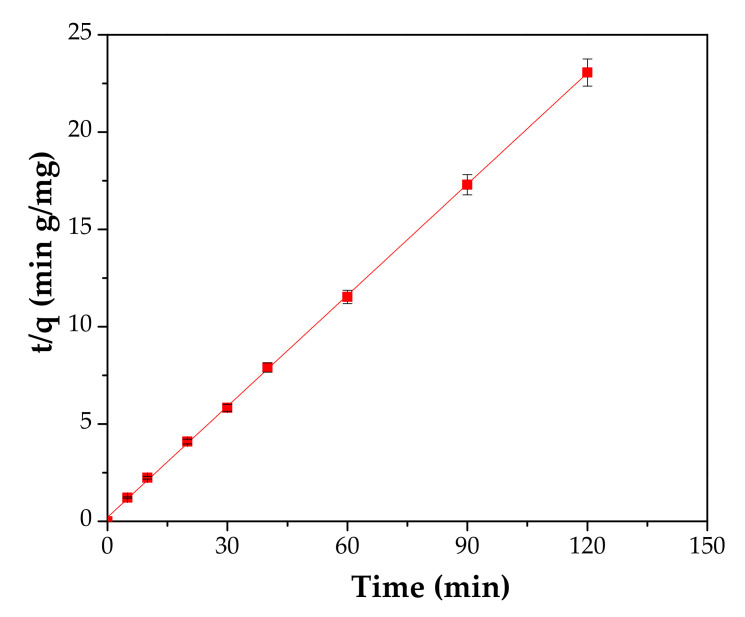
Pseudo-second-order kinetics.

**Figure 13:**
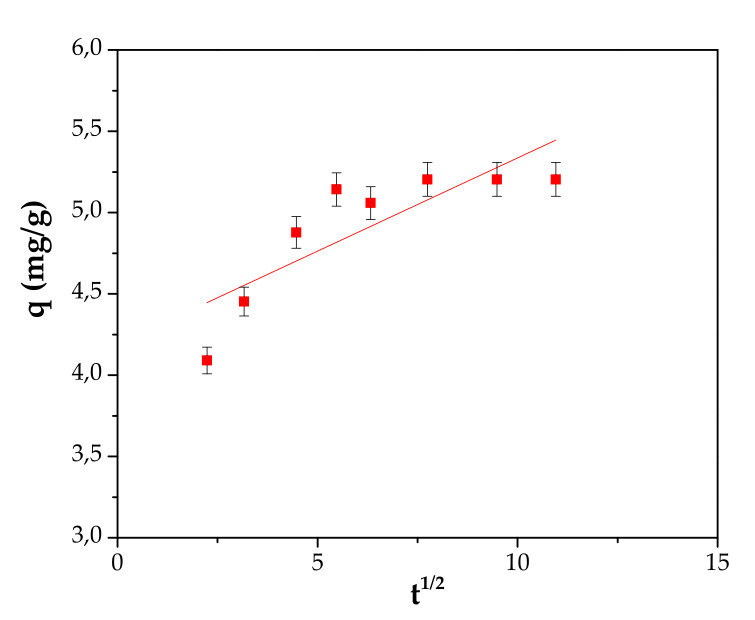
Intraparticle difussion model.

Three kinetic models, the pseudo-first-order model, pseudo-second-order model and intraparticle diffusion model have been developed in order to describe the adsorption kinetic process of Cd^2+^ onto material, see Figure 11, Figure 12 and Figure 13 and Table 4. A detailed presentation for each model can be consulted in the literature [24,40,41].

The linearized form of the pseudo-first-order model is presented as follows:

(3) $$\log\left(q_{e}-q_{t}\right)=\log q_{e}-\frac{\left(k_{1}t\right)}{2.303}$$

The linearized form of the pseudo-second-order model is:

(4) $$\frac{t}{q_{t}}=\frac{1}{k_{2}q_{e}{}^{2}}+\frac{t}{q_{e}}$$

The intraparticle diffusion model is based on Equation (5):

(5) $$q_{t}=k_{i}t^{0.5}$$

where $q_{e}$ is the amount of Cd^2+^ adsorbed at equilibrium (mg/g), $q_{t}$ is the amount of Cd^2+^ adsorbed at time t (mg/g), $k_{1}$ and $k_{2}$ are the reaction rate constants of the pseudo-first order (1/min) and pseudo-second order (g/mg·min); $k_{i}$ is the intraparticle diffusion rate constant.

For this study, the pseudo-second-order model describes the adsorption of Cd^2+^ onto the adsorbent, with a coefficient regression, R^2^, of 0.9999. The experimental $q_{e}$ value of 5.2 mg/g is in agreement with the $q_{e}$ value of 5.28 mg/g calculated from the pseudo-second-model and the $k_{2}$ constant has a value of 0.1308 g/mg·min.

The results obtained in this study show that this adsorbent can successfully treat the waters contaminated with Cd^2+^ ions (Table 5).

## 4. Conclusions

This study confirmed that the FA/H_2_SO_4_ material could remove Cd^2+^ ions from an aqueous solution with the following main observations:

- The adsorbent dose had an effect on the adsorption process: A higher dose led to a decrease in adsorption capacity.
- Moreover, it was found that the adsorption process is dependent on the initial concentration.
- The adsorption equilibrium was reached after 60 min of contact time.
- The data fitted in the Langmuir model with a maximum adsorption capacity of 28.09 mg/g. The adsorption process could be explained through a pseudo-second-order kinetic model. This suggests the dominance of chemisorption and monolayer adsorption.
- The adsorption study demonstrated that the material is an effective adsorbent for the removal of cadmium ions from aqueous solutions.

The use of this type of adsorbent could solve the associated environmental and health effects.

## Figures and Tables

**Table 1:** Elemental composition of FA/H_2_SO_4._

Element	Mass, %
O	59.82826
Si	20.07406
Al	8.967165
Ca	2.286939
Fe	1.500967
K	1.039111
Mg	0.872161
Na	0.638949
Ti	0.344842
S	4.447546

**Table 2:** Elemental composition of FA/H_2_SO_4_+Cd^2+^.

Element	Mass, %
O	53.8552
Si	26.6605
Al	10.7206
Ca	0.7185
Fe	1.8664
K	1.7000
Mg	0.5995
Na	0.5135
Ti	0.3448
Cd	3.0207

**Table 3:** Adsorption isotherm parameters of Cd^2+^ onto FA/H_2_SO_4_.

Langmuir Model $\frac{C_{e}}{q_{e}}=\frac{1}{K_{L}q_{max}}+\frac{C_{e}}{q_{max}}$			Freundlich Model $\log q_{e}=\left(\frac{1}{n}\right)\log q_{e}+\log K_{F}$		
$q_{max}$	$K_{L}$	*R* ^2^	$K_{F}$,	*1/n*	*R* ^2^
28.09	0.0110	0.9956	6.23	0.2101	0.9806

$q_{max\;}$ is the maximum adsorption capacity (mg/g); $K_{L}$ is Langmuir constant (L/g); $K_{F}$ is the Freundlich constant; 1/n is the heterogeneity factor; $\;q_{e}$ is the amount of heavy metal adsorbed at equilibrium (mg/g); $C_{e}$ is the concentration at equilibrium (mg/L).

**Table 4:** Kinetic parameters of Cd^2+^ adsorption onto the FA/H_2_SO_4_ sample.

Kinetic model	Parameters	Values
Pseudo-first order	$k_{1}$, 1/min	0.071
	*R* ^2^	0.763
Pseudo-second order	q_e cal_, mg/g	5.28
	$k_{2}$, g/mg·min	0.1308
	R^2^	**0.9999**
Intraparticle diffusion	$k_{i}$, mg/g·min^0.5^	0.114
	R^2^	0.693

**Table 5:** Comparison of maximum Cd^2+^ adsorption capacities (q_max_) of different adsorbents reported in literature.

Adsorbent	q_max_ (mg/ g)	References
Iranian natural zeolite	4.01	[42]
Modified fly ash	43.12	[25]
Bottom ash	13.70	[37]
Coated Industrial Waste Fly Ash	6.39	[43]
NaOH modified ash	31.79	[28]
Swine manure biochar	46.5	[44]
USA clinoptilolite-K	24.5	[45]
RSA clinoptilolite-K	20.73	[45]
Palm Oil Fuel Ash	10.56	[46]
Fe_3_O_4_@PDA	21.58	[14]
Ceramsite/C-A-S-H/TCPS	14.27	[47]
Fe_3_O_4_@Z	19.9	[34]
***FA/H_2_SO_4_***	***28.09***	***This study***
